# Clinical Outcomes and Molecular Predictors of Pembrolizumab (Keytruda) as a PD-1 Immune Checkpoint Inhibitor in Advanced and Metastatic Cervical Cancer: A Systematic Review and Meta-Analysis

**DOI:** 10.3390/biomedicines12051109

**Published:** 2024-05-16

**Authors:** Lavinia Balan, Anca Maria Cimpean, Prashant Sunil Nandarge, Bogdan Sorop, Catalin Balan, Madalina Alexandra Balica, Felix Bratosin, Simona Brasoveanu, Madalina Boruga, Laurentiu Pirtea

**Affiliations:** 1 Department of Obstetrics and Gynecology, Victor Babes University of Medicine and Pharmacy, 300041 Timisoara, Romania; lavinia.balan@umft.ro (L.B.); bogdansorop@yahoo.com (B.S.); simona.brasoveanu@umft.ro (S.B.); pirtea.laurentiu@umft.ro (L.P.); 2 Doctoral School, Department of General Medicine, University of Medicine and Pharmacy Victor Babes Timisoara, 300041 Timisoara, Romania; bcatalin43@yahoo.com (C.B.); madalina.balica@umft.ro (M.A.B.); felix.bratosin@umft.ro (F.B.); 3 Department of Microscopic Morphology/Histology, Victor Babes University of Medicine and Pharmacy, 300041 Timisoara, Romania; acimpeanu@umft.ro; 4 Center of Expertise for Rare Vascular Disease in Children, Louis Turcanu Children Hospital, 300011 Timisoara, Romania; 5 Department of General Medicine, D.Y. Patil Medical College Kolhapur, Kolhapur 416005, India; prashantnandarge1997@gmail.com; 6 Department of Cellular and Molecular Biology, Victor Babes University of Medicine and Pharmacy, 300041 Timisoara, Romania; 7 Department of Infectious Disease, Victor Babes University of Medicine and Pharmacy, 300041 Timisoara, Romania; 8 Department of Toxicology, Drug Industry, Management and Legislation, Faculty of Pharmacology, Victor Babes University of Medicine and Pharmacy Timisoara, 300041 Timisoara, Romania

**Keywords:** oncology, gynecology, cervical cancer, systematic review

## Abstract

This systematic review evaluates the clinical outcomes and molecular predictors of response to pembrolizumab in patients with advanced and metastatic cervical cancer. We adhered to the PRISMA guidelines for systematic reviews, conducting a database search in PubMed, Scopus, and Embase. The eligibility criteria centered on clinical outcomes, including the overall survival (OS), progression-free survival (PFS), and immune-related biomarkers post-pembrolizumab therapy. We included both prospective and retrospective studies that detailed clinical outcomes and molecular characteristics predictive of therapeutic response. Our search yielded six studies involving 846 patients treated with pembrolizumab from 2017 to 2022. The meta-analysis of these studies showed that pembrolizumab, used as monotherapy or in combination with chemotherapy, extended the OS by a weighted median of 10.35 months and the PFS by 8.50 months. The treatment demonstrated a pooled objective response rate (ORR) of 22.39%, although the I^2^ test result of 67.49% showed a high heterogeneity among the studies. Notably, patients with high PD-L1 expression (CPS ≥ 10) experienced improved outcomes in terms of the PFS and OS. The most common complications were fatigue, diarrhea, and immune-related adverse events. Pembrolizumab significantly enhances clinical outcomes in metastatic cervical cancer, particularly among patients with high PD-L1 expression. The drug maintains a good safety profile, reinforcing its treatment potential for patients with advanced and metastatic cervical cancer. Future studies should explore long-term effects and strategies to integrate pembrolizumab optimally into current treatment regimens, aiming to maximize patient benefits and effectively manage side effects.

## 1. Introduction

Cervical cancer is the seventh most common type of cancer globally, being treatable in the early stages but particularly difficult to diagnose early in settings with limited access to screening and early treatment modalities [1,2,3,4]. As the fourth most common cancer among women worldwide, its burden is exacerbated in the metastatic stage, where prognosis drastically worsens and treatment options become limited [5,6,7,8]. The standard of care for metastatic cervical cancer typically includes chemotherapy, radiation, and surgery, but the overall survival rates remain low, underscoring the urgent need for innovative therapeutic approaches [9,10].

Immune checkpoint inhibitors (ICIs) have emerged as a new class of therapeutics in oncology, offering hope for many types of cancers that were previously deemed refractory to existing treatments, such as monoclonal antibodies [11,12]. Pembrolizumab, a monoclonal antibody targeting the programmed death receptor-1 (PD-1), has shown promising results in several types of cancers [13,14]. The molecular basis for the effectiveness of pembrolizumab hinges on its type as a selective IgG4 kappa monoclonal antibody, with the ability to interfere with the PD-1 pathway, a critical immune checkpoint that cancer cells exploit to evade immune detection [14,15]. In metastatic cancer, the expression of PD-L1, which binds to PD-1, has been correlated with disease progression and prognosis, suggesting a potentially pivotal role for PD-1/PD-L1 inhibitors in altering disease outcomes [16].

Despite the growing interest in pembrolizumab, the landscape of clinical research focusing on its use in metastatic cervical cancer is complex and evolving [17]. Various studies have reported on its safety and efficacy in different types of cancer, but results vary widely, influenced by factors such as tumor genetic profile, prior treatment history, and patient immune status [18].

The objectives of this systematic review are to evaluate the clinical efficacy and molecular insights of pembrolizumab as an immune checkpoint inhibitor in the treatment of metastatic cervical cancer. Specifically, this review aims to (1) assess the impact of pembrolizumab on the overall survival and progression-free survival in patients with metastatic cervical cancer, (2) analyze the molecular characteristics that predict response to treatment with pembrolizumab, and (3) review the safety profile and quality-of-life outcomes for patients treated with this immune checkpoint inhibitor. Through this comprehensive analysis, this review intends to clarify the role of pembrolizumab in enhancing clinical practice and patient care in advanced and metastatic cervical cancer.

## 2. Materials and Methods

### 2.1. Eligibility Criteria 

This review considered studies for the final analysis based on the following inclusion criteria: (1) patients diagnosed with metastatic cervical cancer and treated with pembrolizumab as a monotherapy or in combination with other therapeutic agents; (2) research that explicitly examines the clinical outcomes following the use of pembrolizumab, with particular emphasis on overall survival, progression-free survival, and molecular characteristics predicting responsiveness to therapy; (3) a broad array of study designs, such as randomized controlled trials, observational studies, clinical trials, cohort studies, case–control studies, and cross-sectional studies; (4) studies utilizing validated instruments or clearly defined parameters to assess survival rates, molecular markers of response, adverse events, and quality-of-life outcomes; (5) only peer-reviewed articles published in English, to ensure the feasibility of thorough review and analysis.

The exclusion criteria included the following: (1) research not involving human participants, such as in vitro or animal model studies related to cervical cancer; (2) studies not specifically examining patients with metastatic cervical cancer or those that do not differentiate the impact of pembrolizumab on this specific patient group, along with those that used other ICIs; (3) studies that do not provide clear, quantifiable outcomes related to survival rates, molecular responsiveness, or lack sufficient detail for a comprehensive analysis; (4) grey literature, including non-peer-reviewed articles, preprints, conference proceedings, general reviews, commentaries, and editorials; (5) low-quality studies assessed using quantifiable methods that can determine significant discrepancies in reported data. 

### 2.2. Information Sources

The information sources for the current study were the electronic databases PubMed, Scopus, and Embase. The literature search was targeted to include publications up to 19 February 2024 as the initial search date. The primary objective of the search strategy was to collect studies that evaluated the clinical outcomes, molecular characteristics, patient demographics, and treatment modalities associated with the use of pembrolizumab in treating metastatic cervical cancer. 

### 2.3. Search Strategy

The search strategy utilized the following keywords and phrases pertinent to the study’s objectives: “cervical cancer”, “metastatic cervical cancer”, “metastatic disease”, “advanced cervical cancer”, “pembrolizumab”, “immune checkpoint inhibitors”, “PD-1 inhibitors”, “PD-L1 expression”, “clinical efficacy”, “molecular insights”, “survival outcomes”, “progression-free survival”, “overall survival”, “treatment response”, “adverse effects”, “immunotherapy”, “cancer immunotherapy”, “patient demographics”, “treatment modalities”, and “biomarkers”.

To ensure comprehensive and efficient literature retrieval, Boolean operators (AND, OR, and NOT) were employed to effectively combine and refine these terms along with relevant Medical Subject Headings (MeSH) and keywords. The search string included the following: ((“cervical cancer” OR “advanced cancer” OR “metastatic cervical cancer”) AND (“pembrolizumab” OR “immune checkpoint inhibitors” OR “PD-1 inhibitors”) AND (“clinical efficacy” OR “survival outcomes” OR “treatment response”) AND (“molecular insights” OR “PD-L1 expression” OR “biomarkers”) AND (“overall survival” OR “progression-free survival”) AND (“adverse effects” OR “patient demographics”)).

### 2.4. Selection Process

In accordance with the Preferred Reporting Items for Systematic Reviews and Meta-Analyses (PRISMA) guidelines [19], our selection process involved a structured and transparent method to ensure the reproducibility of our research. Initially, all retrieved records were independently screened by two reviewers to determine their eligibility based on the predefined inclusion and exclusion criteria. Discrepancies between the reviewers were resolved through consultation with a third reviewer. The review protocol is openly accessible on the Open Science Framework (OSF) with the registration code osf.io/tu5k9.

### 2.5. Data Collection Process

The data collection process for this systematic review commenced with the removal of duplicate entries, followed by abstract screening performed by two independent reviewers to assess each study’s relevance based on the predefined inclusion and exclusion criteria. Discrepancies between the reviewers were resolved through discussion or, if necessary, consultation with a third reviewer to achieve consensus. 

### 2.6. Data Items

For this systematic review, we collected data on clinical outcomes related to pembrolizumab treatment for metastatic cervical cancer as defined by the National Comprehensive Cancer Network (NCCN) guidelines [20]. The primary outcomes included overall survival (OS) and progression-free survival (PFS) at 1 year, 3 years, and 5 years, along with adverse event rates, as these metrics are critical in evaluating the efficacy and safety of cancer therapies (NCCN Guidelines for Cervical Cancer, 2023). The secondary outcomes included response rates and duration of response, utilizing the Response Evaluation Criteria in Solid Tumors (RECIST) for assessment standardization [21].

Study and patient characteristics data were also collected, encompassing study design, geographic location, patient demographics (age, gender), and treatment details (dosing, combination therapies). Biomarker data, particularly PD-L1 expression, were included due to their predictive value in response to pembrolizumab, following FDA guidance on biomarker inclusion in clinical trials.

Metastatic cervical cancer was defined as per the American Joint Committee on Cancer (AJCC) staging system, focusing on cases where cancer has spread to distant sites, relevant for assessing pembrolizumab’s role in advanced disease stages (AJCC Cancer Staging Manual, 8th Edition) [22].

The administration of pembrolizumab was characterized according to its labeled use in the treatment of metastatic cervical cancer and depending on the study protocol of the clinical trials involved, reflecting standard practices outlined in the latest FDA drug approval announcements for oncology drugs.

### 2.7. Risk of Bias and Quality Assessment

Initially, the quality of observational studies was evaluated using the Newcastle–Ottawa Scale [23], which assesses three critical dimensions: the selection of study groups, the comparability of these groups, and the ascertainment of either the exposure or outcome of interest for case–control or cohort studies, respectively. Each study is awarded stars in these categories, cumulating in a score that classifies the study quality as either low, medium, or high.

### 2.8. Synthesis Methods

We integrated the findings from the selected studies through a qualitative synthesis, given the variability in the study designs and the outcome measures reported. To prepare the data for synthesis, we performed tabulation of survival outcomes, surgical success rates, and complication rates, while handling missing data by noting their absence and acknowledging potential impacts on our findings. The results from individual studies were summarized and presented descriptively, comparing the survival outcomes and treatment effectiveness.

A meta-analysis was conducted to evaluate the one-year survival rates of patients undergoing R0 resections for locally advanced gastric cancer. We utilized a random-effects model to account for the expected variability across different studies. Survival rates were treated as proportions, and inverse variance weights were calculated for each study to determine a weighted mean survival rate. The between-study variance (T²) was estimated using the DerSimonian method, which adjusts the weights of individual studies to incorporate both within-study and between-study variance. Heterogeneity among the study results was quantified using the I² statistic, which describes the percentage of the total variation across studies that is due to heterogeneity rather than chance. A high I^2^ value indicates substantial variability among the studies. 

## 3. Results

### 3.1. Study Selection and Study Characteristics

**Figure 1:**
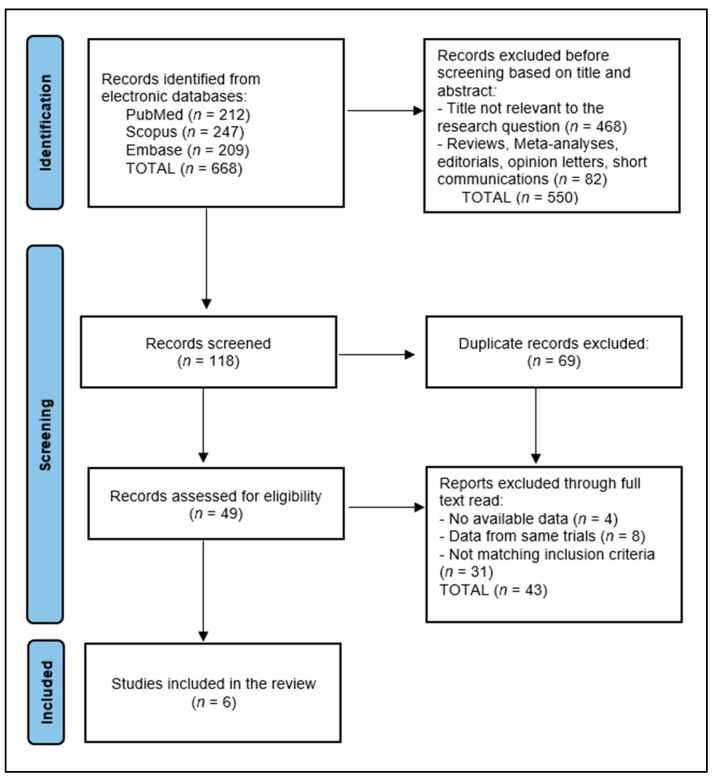
PRISMA flow diagram.

A total of 668 articles were identified according to the initial search, of which 69 duplicate entries were eliminated, 550 records were excluded before screening based on their titles and abstracts, and 43 articles were excluded after a full read for not matching the inclusion criteria or having no available data. The systematic review included a total of six studies in the final analysis, delineated in Figure 1, spanning a period from 2017 to 2024.

The analysis of the characteristics from six studies [24,25,26,27,28,29] on the use of pembrolizumab in recurrent and metastatic cervical cancer, detailed in Table 1, covered a period from 2017 to 2022. These studies predominantly employed randomized clinical trial designs across various phases, with five out of six studies (Frenel et al. [24], Chung et al. [25], Youn et al. [26], Colombo et al. [27], and Nishio et al. [29]) conducted as international trials. The trial phases varied, with Frenel et al. [24] conducting a phase Ib trial in 2017, introducing pembrolizumab’s potential efficacy and safety profile in early clinical evaluation. This was followed by two phase II trials by Chung et al. [25] in 2019 and Youn et al. [26] in 2020, each further exploring the therapeutic implications and refining the clinical applications of pembrolizumab. Subsequent phase III trials by Colombo et al. [27] in 2021 and Nishio et al. [29] in 2022 significantly contributed to establishing a more comprehensive understanding of the drug’s efficacy and safety in a larger patient population. In contrast, the study by Miller et al. [28] in 2021 adopted a retrospective cohort design. 

### 3.2. Results of Individual Studies

The studies [24,25,26,27,28,29] varied considerably in their sample size and follow-up durations, providing a broad spectrum of data. Frenel et al. [24] enrolled 24 patients, with a median age of 42 years and a median follow-up time of 11.0 months. The performance status indicated a majority of patients (75%) had an ECOG score of 1, suggesting moderate functional impairment. Chung et al. [25] included a larger cohort of 98 patients with a median age slightly higher at 46 years. The median follow-up time was 10.2 months, and similarly to Frenel et al., a large proportion of the patients (65.3%) had an ECOG status of 1. This study also lacked a comparison group, focusing solely on the outcomes for pembrolizumab-treated patients.

Youn et al. [26] studied 36 patients over a shorter median follow-up of 6.2 months, with the patients being older, having a median age of 51 years. The balance in performance status was more even, with 53% of patients scoring ECOG 0, indicative of better overall functioning compared to the other studies. Colombo et al. [27] conducted the largest study with 617 patients, divided almost equally between the pembrolizumab (308) and placebo (309) arms, alongside chemotherapy. The median follow-up was notably longer at 22.0 months, with a median age of 50 years.

Miller et al. [28] presented data from a small sample of 14 patients, with a median overall survival of 11.2 months and a follow-up of 14.4 months. The median age was the highest among the studies at 59 years. Lastly, Nishio et al. [29] included 57 patients, split between pembrolizumab (35) and placebo (22) groups. The follow-up time was extensive, at 23.2 months, with a median age of 54 years. The performance status was better in the pembrolizumab group, with 83% having an ECOG score of 0 compared to 73% in the placebo group (Table 2).

### 3.3. Results of Synthesis

Frenel et al.’s study [24] involved patients predominantly in the metastatic stages (M1: 63%), with the majority having squamous cell carcinoma (96%). Nearly all the patients were PD-L1-positive (100%), reflecting a selection toward likely responders to pembrolizumab. Prior treatments were heavily weighted toward radiotherapy (92%) and platinum-based therapies (96%). Chung et al. [25] reported on patients almost exclusively in stage IVB (93.9%), with a high prevalence of squamous cell carcinoma (93.9%). PD-L1 positivity was noted in 83.7% of the patients, and all had received prior chemotherapy, with a significant proportion also treated with bevacizumab (41.8%) and radiotherapy (86.7%).

Youn et al. [26] focused solely on advanced-stage patients, with a substantial percentage having adenocarcinoma (22%). This study also detailed HPV involvement, with HPV-16 being predominant (72%). PD-L1 positivity was significant (72%), and the patients varied in their prior treatments, with many having received multiple lines of therapy. Colombo et al. [27] presented a complex array of stages ranging from III to IVB, with a notable focus on squamous cell carcinoma (76.3%). The PD-L1 combined positive score (CPS) showed that more than half of the patients had high PD-L1 expression (CPS ≥ 10: 51.3%). Treatments prior to the study included a combination of chemoradiotherapy, surgery, and radiotherapy.

Miller et al. [28] had a mixed-stage group (III and IV), with diverse histology, including squamous cell carcinoma and variants of adenocarcinoma. PD-L1 CPS >1% was observed in the vast majority (93%), and almost all the patients had prior radiotherapy, with varying lines of prior chemotherapy. Lastly, Nishio et al. [29] differentiated their patient cohort based on their initial treatment arms, showing a stark contrast in stage IVB presence between the pembrolizumab (31%) and placebo groups (64%). PD-L1 CPS varied widely, with similar proportions across the lower and higher expression levels in both treatment groups. Prior treatment histories were diverse, with many in the pembrolizumab group having not received any prior treatment (11%), unlike the placebo group (45%), as seen in Table 3.

Frenel et al. [24] treated patients with a relatively high dose of pembrolizumab (10 mg/kg every 2 weeks) for up to 24 months. The median follow-up time was 11 months, during which an ORR of 17% was observed, with a median PFS of 2 months and median OS of 11 months. The study concluded that pembrolizumab was effective and well tolerated, aligning with its safety profile in other tumor types. Chung et al. [25] administered pembrolizumab at a fixed dose of 200 mg every 3 weeks for up to 2 years, achieving a slightly lower ORR of 12.2%, and 14.6% specifically in the PD-L1-positive patients. The median PFS and OS were 2.1 months and 9.4 months, respectively. These outcomes led to the FDA’s accelerated approval for pembrolizumab in PD-L1-positive advanced cervical cancer cases, highlighting its durable antitumor activity and manageable safety profile.

Youn et al. [26] explored a combination therapy of GX-188E and pembrolizumab, which resulted in a higher ORR of 42% at 24 weeks. The median OS was recorded at 10.2 months, with a 6-month PFS of 35%, suggesting the combination therapy’s potential as a new treatment option for this patient population. Colombo et al. [27] combined pembrolizumab with chemotherapy (and optionally bevacizumab), reporting a median follow-up of 22 months. The median PFS was 10.4 months, with an OS 24-month estimate of 53.0% in the pembrolizumab group versus 41.7% in the placebo group. This combination significantly improved PFS and OS, affirming the therapeutic benefit of adding pembrolizumab to standard chemotherapy in patients with PD-L1 CPS ≥ 1.

**Figure 2:**
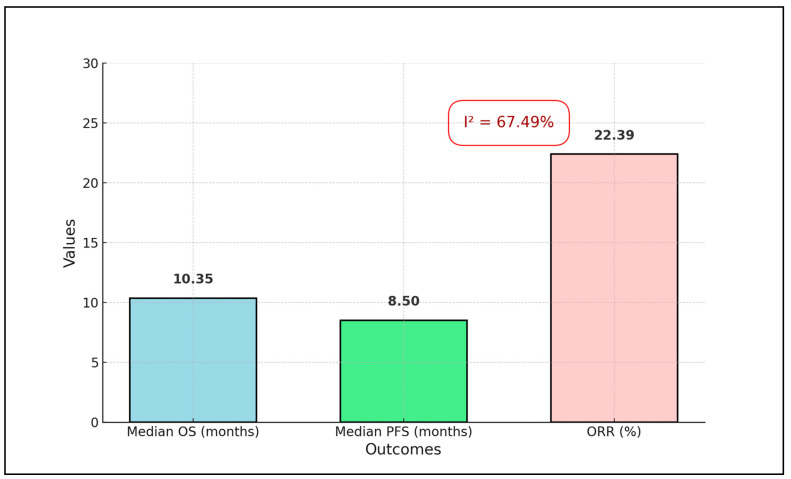
Weighted average survival metrics across the analyzed studies.

Miller et al. [28] also used 200 mg of pembrolizumab every 3 weeks but in heavily pretreated patients, leading to an ORR of 21%. Although the median PFS was not specified, the median OS was 11.2 months. The study emphasized pembrolizumab’s efficacy particularly in patients with limited metastatic sites and high tumor mutational burden (TMB). Nishio et al. [29] administered pembrolizumab alongside chemotherapy and optionally bevacizumab, achieving impressive results with the median PFS and OS not yet reached; however, the hazard ratios (HRs) for PD-L1 CPS ≥ 1 were significantly favorable (PFS HR: 0.36, and OS HR: 0.38), as presented in Table 4. This indicates a substantial prolongation of PFS and OS compared to placebo plus chemotherapy, supporting pembrolizumab’s use in combination with chemotherapy as an effective treatment modality in this subgroup. Overall, the pooled objective response rate was 23.27%, the progression-free survival was 12.17%, while the overall survival was 17.24%, as presented in Figure 2.

**Figure 3:**
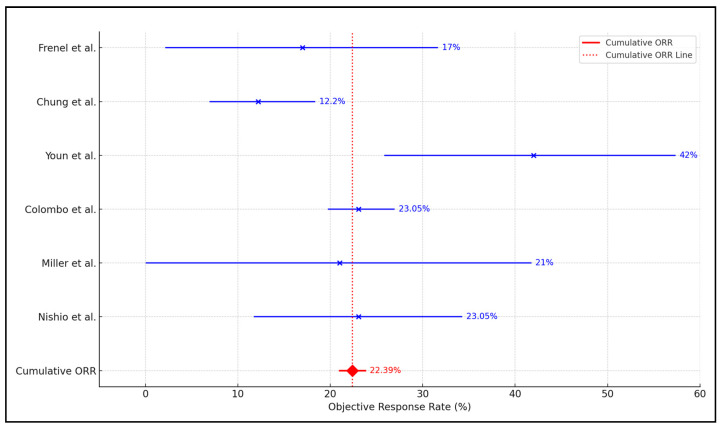
Forest plot analysis of objective response rate [24,25,26,27,28,29].

The meta-analysis found a weighted average of 10.35 months for OS, 8.50 months for PFS, and 22.39% for ORR. A significant portion of the analysis’ weight was attributed to larger studies, such as Colombo et al., which represented approximately 72.9% of the overall weight, emphasizing the influence of large-scale studies on the aggregated results. The heterogeneity among the included studies was considerable, with an I² value of approximately 67.49% (Figure 2), indicating substantial variability in the treatment effects across different study conditions and populations. This high level of heterogeneity highlights the impact of diverse factors, such as patient demographics, disease stages, and specific treatment protocols on the outcomes, underscoring the need to interpret the pooled estimates with consideration of the underlying study variations, as presented in Table 5 and Figure 3.

## 4. Discussion

### 4.1. Summary of Evidence

The current findings consolidate pembrolizumab’s efficacy in recurrent and metastatic cervical cancer. A noteworthy observation from the analyzed studies [24,25,26,27,28,29] is the consistent evidence of pembrolizumab’s antitumor activity, particularly in PD-L1-positive patients, demonstrated from the survival outcomes and objective response rates reported, which, despite varying across the studies, generally support the use of pembrolizumab for this subset of patients. For instance, Chung et al. [25] and Nishio et al. [29] provided compelling evidence leading to FDA accelerated approval, that further reinforces pembrolizumab’s efficacy and safety.

However, the results also underscore the challenges associated with pembrolizumab treatment, such as the relatively short median progression-free survival observed in several studies, like those by Frenel et al. [24] and Chung et al. [25], where the median PFS was reported at just around 2 months. This indicates a need for further investigation into how pembrolizumab can be better integrated into treatment regimens, possibly in combination with other therapies, to enhance its effectiveness and extend PFS. The combination strategies illustrated by Colombo et al. [27] and Youn et al. [26], which integrate chemotherapy and novel therapeutic agents like GX-188E with pembrolizumab, suggest a promising direction that could potentially amplify clinical benefits.

The studies by Tewari et al. [30] and Lin Qi et al. [31] present insightful contrasts and similarities regarding the efficacy of pembrolizumab in treating cervical cancer, particularly highlighting its performance across different patient subgroups and general populations. Tewari et al.’s trial underscores the enhanced efficacy of pembrolizumab when added to chemotherapy with or without bevacizumab, showing favorable hazard ratios for overall survival across various subgroups, such as those using bevacizumab (HR 0.62) and carboplatin (HR 0.65), as well as in both squamous (HR 0.60) and nonsquamous histologies (HR 0.70). However, the study was not included in the final analysis of this systematic review for reporting on the same KEYNOTE-826 trial as Frenel et al. [24]. Conversely, Lin Qi et al.’s meta-analysis focusing on the safety and efficacy of pembrolizumab in all categories of cervical cancer reported more modest outcomes, with an objective response rate of 15.5% and a disease control rate of 33.1%, alongside a median OS and progression-free survival of 10.23 months and 4.27 months, respectively. 

Other studies provided insights into the effectiveness of pembrolizumab monotherapy in recurrent cervical cancer, illustrating its application in both a smaller, focused cohort and a broader, real-world setting, respectively. Gen et al. [32] reported a higher objective response rate of 28.6% among 14 patients, with one complete response and three partial responses, highlighting modest antitumor activity without any treatment discontinuations or related deaths. In contrast, Choi et al.’s [33] larger study across sixteen institutions involving 117 patients showed a lower ORR of 9.4%, with three complete responses and eight partial responses. However, they noted a significant variance in efficacy depending on the patients’ performance status, where those with a favorable status (ECOG ≤ 1) experienced a higher ORR of 18.9%. Moreover, while Gen et al. observed no severe adverse events, Choi et al. reported grade ≥3 adverse events in 6.8% of their cohort, including two suspected treatment-related deaths, indicating a need for the careful monitoring of safety profiles in a larger, diverse patient population. Both studies underscore pembrolizumab’s potential, yet they also reflect the challenges of predicting outcomes across different clinical settings.

Smaller studies, like the case reports by Hsiu-Jung Tung et al. [34] and Mengmeng Lyu et al. [35], explore the efficacy of pembrolizumab combined with chemoradiotherapy in treating advanced and recurrent cervical cancer, each offering valuable insights with varying degrees of success and complications. Tung et al. report on three cases where complete remission was achieved through the integration of pembrolizumab with chemoradiotherapy and, in one instance, proton therapy. Each patient had stage IVB cervical cancer with different histologies and recurrence sites, but all responded favorably to the treatment combination, suggesting a potentially curative approach for conventionally palliative situations. In contrast, Lyu et al. presented a single case of stage IVB cervical cancer treated with chemotherapy (albumin-bound paclitaxel and carboplatin) combined with pembrolizumab, followed by radiotherapy. Although the patient initially saw almost complete resolution after six cycles, severe grade 4 myelosuppression forced a cessation of chemotherapy and radiotherapy, leading to explosive tumor growth. 

In their study, Ngoi N.Y.L. et al. [36] explored the molecular characteristics of tumors from patients treated with pembrolizumab monotherapy, focusing on metastatic cervical cancer cases that were resistant to first-line chemotherapy. Through immunohistochemistry and next-generation sequencing, they found notable genetic insights: the patient with a prolonged partial response, despite lacking tumoral PD-L1 expression, showed a PD-L1 CPS of 1 and had mutations in ERBB4, PIK3CA, and RB1. Meanwhile, other patients, despite disease progression, had PD-1 expression in stromal lymphocytes and shared a PIK3CA mutation, suggesting that these molecular markers might influence the response to pembrolizumab.

Other studies analyzed the cost-effectiveness of pembrolizumab combined with chemotherapy in treating recurrent or metastatic cervical cancer, each presenting insights from different perspectives and outcomes. Zheng et al. [37] found that adding pembrolizumab to chemotherapy for patients with a PD-L1 CPS of >1 yielded an incremental cost-effectiveness ratio (ICER) of USD 64,338 per QALY, which surpasses China’s willingness-to-pay threshold, suggesting it may not be cost-effective. In contrast, Lin et al. [38] reported a higher ICER of USD 114,275.67 per QALY for the combination of pembrolizumab, chemotherapy, and bevacizumab, also exceeding the acceptable threshold in China, though they noted an improved cost-effectiveness for patients with a higher PD-L1 CPS (≥10). Barrington et al. [39] detailed a more favorable scenario in the U.S. context, where chemotherapy plus pembrolizumab was found to be cost-effective compared to chemotherapy plus bevacizumab, with an ICER of USD 92,678 per QALY; however, the combination of all three treatments was not cost-effective unless pembrolizumab’s price was substantially reduced. These findings reflect varying regional economic evaluations and highlight the influence of PD-L1 expression levels on the cost-effectiveness of pembrolizumab in different healthcare settings.

Nevertheless, it is worth mentioning other studies on the potential of pembrolizumab in cervical cancer, even though they were excluded from the current analysis due to focusing on earlier stages and localized disease. The studies by Duska et al. [40] and Domenica Lorusso et al. [41] both explored the integration of pembrolizumab with chemoradiation for treating locally advanced cervical cancer (LACC), revealing both promising efficacy and notable safety concerns. Duska et al.’s phase 2 study demonstrated the feasibility of adding pembrolizumab either sequentially or concurrently with pelvic chemoradiation. They reported significant treatment-related toxicity, with 88% of patients experiencing grade 2 or higher adverse events (AEs), including 11 instances of grade 4 AEs. Despite the high toxicity, the completion rates for cisplatin and pembrolizumab were high, with 100% and 83%, respectively, in arm 1, and were slightly lower in arm 2. Conversely, Lorusso et al.’s phase 3 trial, a larger and more structured study, showed that pembrolizumab combined with chemoradiotherapy led to an improvement in progression-free survival rates (68% vs. 57% at 24 months) compared to placebo, with a hazard ratio for disease progression or death at 0.70. However, high-grade AEs were also significant here, reported at 75% in the pembrolizumab group versus 69% in the placebo group. Both studies affirm the potential benefits of incorporating pembrolizumab into the treatment regimen for LACC but underscore the necessity of managing considerable adverse effects to optimize patient outcomes. 

Chemotherapy, particularly with platinum-based drugs such as cisplatin, plays a critical role in the management of cervical cancer, improving survival especially in advanced stages of the disease [7]. Research has highlighted significant advancements in the molecular understanding of how these drugs attack cancer cells, enhancing their efficacy and the development of personalized treatment strategies [42]. Studies have also emphasized the importance of integrating chemotherapy with radiotherapy to improve outcomes, showcasing the benefits of this combination therapy in clinical practice [10]. Moreover, ongoing research into new chemotherapeutic agents aims to increase the precision and reduce the side effects of cervical cancer treatments [43]. These findings collectively underscore the necessity of chemotherapy in comprehensive cervical cancer treatment protocols, driving forward both clinical outcomes and therapeutic innovation [41,43].

### 4.2. Limitations

The variability in study designs and patient demographics across the trials presents an analytical challenge but also a unique opportunity to understand the differential impact of pembrolizumab. The studies ranged from single-arm trials to randomized controlled trials with or without placebo controls and included diverse patient populations in terms of previous treatment histories and disease severity. This heterogeneity, while complicating direct comparisons, enriches the understanding of pembrolizumab’s role across different settings and its adaptability to various patient needs.

## 5. Conclusions

In conclusion, this systematic review reveals that while pembrolizumab holds significant promise as a treatment for recurrent and metastatic cervical cancer, especially in PD-L1-positive populations, its real-world efficacy might be optimized through tailored combination therapies and more stratified patient selection. Future research should focus on long-term outcomes, the integration of pembrolizumab into multi-modal treatment approaches, and the exploration of biomarkers that predict response to treatment.

## Figures and Tables

**Table 1:** Study characteristics.

Number	First Author	Reference	Country	Study Year	Registration	Study Design	Study Quality
1	Frenel et al.	[24]	International	2017	NCT02054806	Randomized clinical trial (phase Ib)	High
2	Chung et al.	[25]	International	2019	NCT02628067	Randomized clinical trial (phase II)	High
3	Youn et al.	[26]	International	2020	NCT03444376	Randomized clinical trial (phase II)	High
4	Colombo et al.	[27]	International	2021	NCT03505710	Randomized clinical trial (phase III)	High
5	Miller et al.	[28]	USA	2021	CA190174	Retrospective cohort	High
6	Nishio et al.	[29]	International	2022	NCT03635567	Randomized clinical trial (phase III)	High

**Table 2:** *C*haracteristics of patients.

Number	First Author	Reference	Sample Size	Follow-Up Time/Mean Survival	Age (Years)	Comparison Group	Performance Status
1	Frenel et al.	[24]	24 (pembrolizumab)	Median: 11.0 months (range: 1.3 to 32.2 months)	Median: 42 (range: 26 to 62)	Not applicable (single-arm trial)	ECOG 0: 25%, ECOG 1: 75%
2	Chung et al.	[25]	98 (pembrolizumab)	Median: 10.2 months (range: 0.6 to 22.7 months)	Median: 46 (range: 24 to 75)	Not applicable (single-arm study)	ECOG 0: 34.7%, ECOG 1: 65.3%
3	Youn et al.	[26]	36 (pembrolizumab)	Median: 6.2 months (IQR: 3.5–8.1)	Median: 51	Not applicable (single-arm study)	ECOG 0: 53%, ECOG 1: 47%
4	Colombo et al.	[27]	617 (pembrolizumab: 308; placebo: 309)	Median: 22.0 months (range: 15.1 to 29.4)	Median: 50 (range: 22 to 82)	Pembrolizumab + chemotherapy vs. placebo + chemotherapy	ECOG 0: 57.8%, ECOG 1: 41.6%
5	Miller et al.	[28]	14 (pembrolizumab)	Median OS: 11.2 months; median follow-up: 14.4 months	Median: 59 (range: 22–77)	Not applicable (single-arm trial)	NR
6	Nishio et al.	[29]	57 (pembrolizumab: 35; placebo: 22)	23.2 months (range: 16.4–27.8 months)	Median: 54 (range: 26–82)	Pembrolizumab + chemotherapy vs. placebo + chemotherapy	ECOG 0: 83% in pembrolizumab group, 73% in placebo group; ECOG 1: 17% in pembrolizumab group, 27% in placebo group

NR—not reported; ECOG—Eastern Cooperative Oncology Group; chemotherapy consisted of paclitaxel + cisplatin/carboplatin.

**Table 3:** Disease characteristics.

Number	First Author	Reference	Stage	Histology	Metastases	HPV/PD-L Status	Prior Treatment
1	Frenel et al.	[24]	MX: 1 (4%); M0: 6 (25%); M1: 15 (63%); Unknown: 2 (8%)	Squamous cell carcinoma: 96%; adenocarcinoma: 4%	Lymph nodes: 67%; lung: 38%; pelvis: 38%; liver: 25%	PD-L1-positive: 100%	Prior radiotherapy: 92%; prior platinum: 96%; prior bevacizumab: 42%
2	Chung et al.	[25]	IVB predominant (93.9%)	Squamous cell carcinoma: 93.9%; adenocarcinoma: 5.1%; adenosquamous: 1.0%	NR	PD-L1-positive: 83.7%	Prior chemotherapy: 100%; bevacizumab: 41.8%; radiotherapy: 86.7%
3	Youn et al.	[26]	Advanced stage: 100% (specific staging NR)	Adenocarcinoma: 22%; squamous cell carcinoma: 78%	NR	HPV-16: 72%; HPV-18: 25%; co-infected: 3%; PD-L1-positive: 72%; PD-L1-negative: 28%	Previous radiotherapy: 75%; previous lines of therapy: 1 line (44%); 2 lines (28%); ≥3 lines (19%)
4	Colombo et al.	[27]	III: 1.6%; IIIA: 1.3%; IIIB: 14.9%; IVA: 2.3%; IVB: 30.5%	Adenocarcinoma: 18.2%; adenosquamous carcinoma: 4.9%; squamous cell carcinoma: 76.3%	Metastatic: 18.8%; persistent or recurrent with distant metastases: 64.6%; persistent or recurrent without distant metastases: 16.6%	PD-L1 combined positive score (CPS) status: <1: 11.4%; 1 to <10: 37.3%; ≥10: 51.3%	Chemoradiotherapy and surgery: 15.9%; radiotherapy and surgery: 7.1%; chemoradiotherapy only: 40.6%; radiotherapy only: 10.1%; surgery only: 7.5%; none: 18.8%
5	Miller et al.	[28]	III: 36%; IV: 29%	Squamous cell carcinoma: 79%; endocervical adenocarcinoma: 7%; mixed adenocarcinoma (clear cell + endometrioid): 7%; mesonephric: 7%	Lung only: 21%; lymph node only: 14%; multi-site: 36%; other specific sites: 29%	PD-L1 CPS > 1%: 93% (13/14 patients tested)	Majority had prior radiotherapy: 93%; various lines of chemotherapy ranging from 1 to 4 prior lines
6	Nishio et al.	[29]	IVB: 31% in pembrolizumab group, 64% in placebo group; persistent or recurrent with distant metastases: 69% in pembrolizumab group, 41% in placebo group; persistent or recurrent without distant metastases: 20% in pembrolizumab group, 14% in placebo group	Adenocarcinoma: 17%; adenosquamous carcinoma: 6%; squamous cell carcinoma: 77%	Yes (initial metastatic diagnosis: 11% in pembrolizumab group, 46% in placebo group)	PD-L1 CPS < 1: 14% in pembrolizumab group, 5% in placebo group; PD-L1 CPS 1 to <10: 43% in pembrolizumab group, 50% in placebo group; PD-L1 CPS ≥10: 43% in pembrolizumab group, 46% in placebo group	Various previous therapies: chemoradiotherapy only: 49% in pembrolizumab group, 41% in placebo group; radiotherapy only: 6% in pembrolizumab group, 5% in placebo group; surgery only: 11% in pembrolizumab group, 0% in placebo group; None: 11% in pembrolizumab group, 45% in placebo group

NR—not reported; HPV—human papilloma virus; PD-L1—programmed death ligand.

**Table 4:** Analysis of outcomes.

Number	First Author	Reference	Treatment/Dose	Follow-Up	Survival	Conclusions
1	Frenel et al.	[24]	Pembrolizumab: 10 mg/kg every 2 weeks for up to 24 months	Median: 11.0 months (range: 1.3 to 32.2 months)	ORR: 17% (4 patients achieved PR); median PFS: 2 months; median OS: 11 months	Pembrolizumab demonstrated antitumor activity and was well tolerated in patients with PD-L1-positive advanced cervical cancer, consistent with safety profiles seen in other tumor types.
2	Chung et al.	[25]	Pembrolizumab: 200 mg every 3 weeks for up to 2 years	Median: 10.2 months (range: 0.6 to 22.7 months)	ORR: 12.2% in total, 14.6% in PD-L1-positive; median PFS: 2.1 months; median OS: 9.4 months	Pembrolizumab demonstrated durable antitumor activity and manageable safety in previously treated advanced cervical cancer, leading to FDA accelerated approval for PD-L1-positive cases.
3	Youn et al.	[26]	GX-188E 2 mg IM + pembrolizumab: 200 mg IV every 3 weeks	Median: 6.2 months (range: 3.5–8.1)	24-week ORR: 42%; median OS: 10.2 months; 6-month PFS: 35%	The combination of GX-188E and pembrolizumab showed promising antitumor activity and manageable safety in advanced cervical cancer, offering a new potential treatment option for this patient population.
4	Colombo et al.	[27]	Pembrolizumab: 200 mg every 3 weeks for up to 35 cycles + chemotherapy ± bevacizumab	Median follow-up: 22.0 months	PFS: median: 10.4 months in the intention-to-treat population, HR 0.65; OS: 24-month estimate, 53.0% in pembrolizumab group vs. 41.7% in placebo group	Pembrolizumab plus chemotherapy significantly improved PFS and OS compared to placebo plus chemotherapy in patients with PD-L1 CPS ≥ 1, demonstrating an effective and manageable safety profile.
5	Miller et al.	[28]	Pembrolizumab: 200 mg every 3 weeks	Median follow-up: 14.4 months	ORR: 21% (3/14); DCB: 36%; median PFS not specified; median OS: 11.2 months	Pembrolizumab demonstrated activity in heavily pretreated patients with advanced cervical cancer, especially beneficial in patients with limited metastatic sites (lung/lymph node only) and high TMB.
6	Nishio et al.	[29]	Pembrolizumab: 200 mg Q3W for up to 35 cycles + chemotherapy (paclitaxel 175 mg/m^2^ + cisplatin 50 mg/m^2^ or carboplatin AUC 5) with or without bevacizumab 15 mg/kg	Median follow-up: 23.2 months	PFS (PD-L1 CPS ≥ 1): HR 0.36 (95% CI, 0.16–0.77), median not reached; OS (PD-L1 CPS ≥ 1): HR 0.38 (95% CI, 0.14–1.01), median not reached	Pembrolizumab plus chemotherapy significantly prolonged PFS and OS versus placebo plus chemotherapy in patients with PD-L1 CPS ≥ 1, demonstrating an effective and manageable safety profile.

NR—not reported; CI—confidence interval; PD-L—programmed death ligand; OS—overall survival; PFS—progression-free survival.

**Table 5:** Meta-analysis.

Number	First Author	Reference	Sample Size	Median OS (Months)	Median PFS (Months)	ORR (%)	Weight
1	Frenel et al.	[24]	24	11	2	17	0.028
2	Chung et al.	[25]	98	9.4	2.1	12.2	0.116
3	Youn et al.	[26]	36	10.2	4.83	42	0.043
4	Colombo et al.	[27]	617	10.45	10.4	23.05	0.729
5	Miller et al.	[28]	14	11.2	4.83	21	0.017
6	Nishio et al.	[29]	57	10.45	4.83	23.05	0.067

NR—not reported; ORR—objective response rate; OS—overall survival; PFS—progression-free survival.

## Data Availability

The original contributions presented in this study are included in the article; further inquiries can be directed to the corresponding author.
